# Epithelioid angiosarcoma of the adrenal gland with metastasis: a case report and literature review

**DOI:** 10.1097/MS9.0000000000000789

**Published:** 2023-05-10

**Authors:** Mahmoud Noman, Almotazbellah M.M Zeer, Zahraa M.M. Zeer, Raed Daas, Yousef Hamamra

**Affiliations:** a Faculty of Medicine, Al-Quds University; b Augusta Victoria Hospital, Jerusalem, Palestine

**Keywords:** adjuvant therapy, adrenal gland, angiosarcoma, case report, immunohistochemistry

## Abstract

Primary adrenal epithelioid angiosarcoma is an exceptionally rare condition, with only 51 cases reported in the literature since 1988.

**Case presentation:**

We report a case of a 59-year-old male patient who presented with a 4-month history of left flank pain and anemia. Radiographic imaging identified a 14 cm mass lesion in the left suprarenal region, which showed heterogeneous enhancement. The patient underwent total adrenalectomy with tumor-free surgical margins. Histological sections showed features consistent with an epithelioid tumor, and immunohistochemical staining confirmed the diagnosis of epithelioid angiosarcoma of the left adrenal gland.

**Discussion:**

Primary adrenal epithelioid angiosarcoma is a very rare entity. It was first described by Kareti *et al.* in 1998. The most common presentation is an abdominal mass associated with pain. As there are no specific imaging findings for this tumor, histology combined with immunohistochemistry is the most definitive diagnostic method. Surgery with adjuvant chemotherapy is the management reported for previous cases.

**Conclusion:**

In cases of rare malignancies, interdisciplinary collaboration is crucial for determining the optimal management strategy.

## Introduction

Highlights

- Angiosarcoma is a very rare and aggressive malignancy. Visceral type is rare than skin and superficial soft tissues, with only 51 reported cases in the adrenal gland since 1988.
- The most definitive diagnosis for such rare malignancies is histology and immunohistochemistry.
- Definitive management is controversial, but in the reported cases, it involved surgical resection with adjuvant chemotherapy if complete eradication is not assured.
- The etiology of adrenal angiosarcoma is not known, but it has been linked to many chemicals like vinyl chloride.

With the improvement in imaging technologies, incidental adrenal mass has become a more frequent finding, with a prevalence of 0.4–5% in computed tomography (CT) imaging and ~8% in autopsy^1^. It is essential to distinguish benign from malignant as well as functioning from nonfunctioning masses. Most of these masses are reported to be benign adenomas, with a prevalence of 80%. However, metastatic malignancies are reported more frequently than primary adrenal malignancies, with a prevalence of 19%^2^.

Adrenal epithelioid angiosarcoma is a rare malignant neoplasm that accounts for less than 1% of all sarcomas. It primarily occurs in the skin and soft tissues, with less prevalence in visceral and internal organs^3^. Primary adrenal epithelioid angiosarcoma is an extremely rare entity with an unknown etiology, although it has been linked to exposure to various carcinogens such as arsenic and vinyl chloride^4^. Due to the rarity of this disease and the limited documentation of previous cases in the literature, diagnosis and treatment present significant challenges^5^. In this case report, we present a new case of primary adrenal epithelioid angiosarcoma in order to add to the existing scientific knowledge of this entity.

This case was written according to the SCARE (Surgical CAse REport) criteria 2020^6^.

## Case presentation

A 59-year-old male patient with a medical history of hypertension and type 2 diabetes mellitus presented with a left adrenal mass. The patient reported experiencing dull pain in the left flank area, weight loss, and fatigue for a period of 4 months. There was no reported family history of malignancy, and the patient denied experiencing headaches, diaphoresis, bouts of hypertension, nervousness, palpitations, diarrhea, vomiting, change in bowel habits, flushing, skin changes, or any other complaints. The patient is employed as a teacher with no occupational exposure to toxic substances, alcohol, or tobacco. Upon evaluation, the patient’s hemoglobin level was found to be low at 7.5, compared to 11 g/dl 8 weeks prior to presentation.

**Figure 1:**
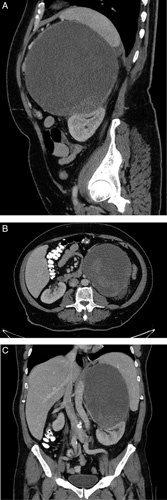
Sagittal, axial, and coronal computed tomography (CT) images showing heterogenous left apical mass. Sagittal (A), axial (B), and coronal (C) CT images show a solid tumor measuring 17 cm in diameter at the upper pole of the left kidney, with peripheral calcifications. It was found to be abutting the spleen and displacing the left kidney inferiorly, as well as the tail of the pancreas anteriorly.

CT revealed the presence of a solid tumor measuring 17 cm in diameter at the upper pole of the left kidney, with peripheral calcifications (Fig. 1). The tumor was found to be abutting the spleen and displacing the left kidney inferiorly, as well as the tail of the pancreas anteriorly. The imaging did not show any evidence of free fluid, regional, or paraaortic lymphadenopathy. The radiographic features of the adrenal lesion were consistent with primary adrenal carcinoma, and there was no indication of distant metastasis. Serum levels of aldosterone, renin, cortisol, testosterone, DHEA-S (dehydroepiandrosterone sulfate), estradiol, androstenedione, 17-OH progesterone (17-hydroxyprogesterone), metanephrine, and normetanephrine were within normal limits.

**Figure 2:**
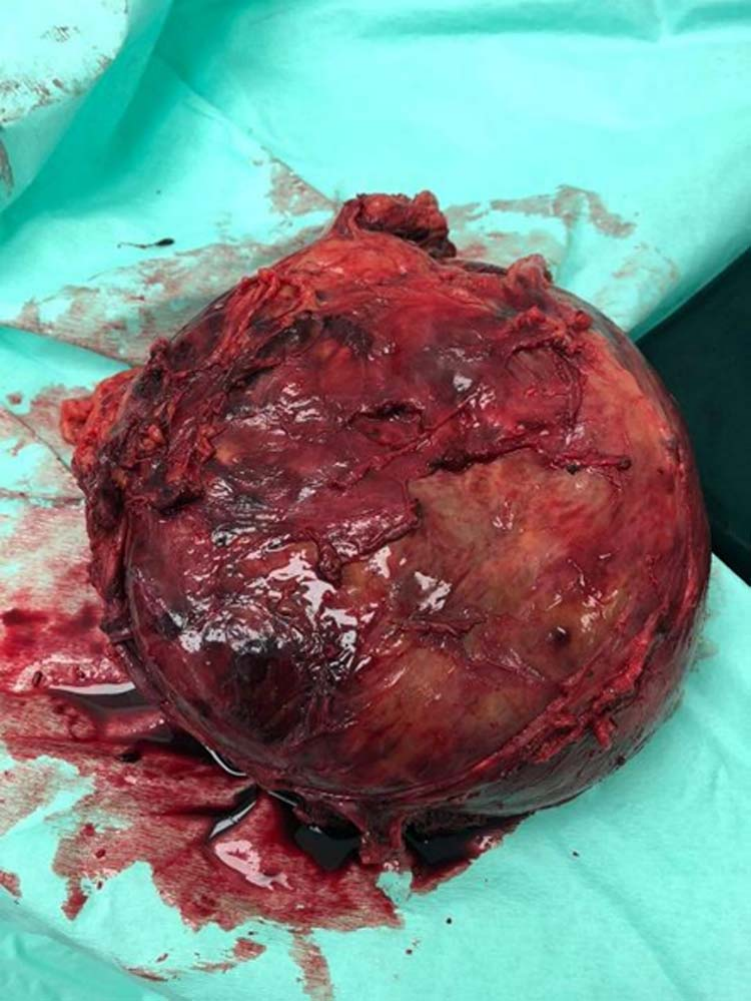
Macroscopic examination. An encapsulated mass involving the entire adrenal gland measuring 14×13×10 cm. Step-sectioning revealed a friable cut surface and grayish-white areas with regions of hemorrhage and necrosis.

The patient underwent a total adrenalectomy procedure with negative surgical margins. An open radical adrenalectomy was performed, and a hypervascular retroperitoneal mass measuring 20×20 cm was excised through a subcostal intraperitoneal incision (Fig. 2). Despite its adherence to adjacent structures such as the stomach, pancreas, spleen, and blood vessels, the mass could be dissected from these structures without causing any damage. As the tumor did not invade the left kidney, only Gerota’s fascia was removed along with the tumor. No residual tumor tissue was detected during the surgery. The tumor was demarcated from the left kidney and the tail of the pancreas.

**Figure 3:**
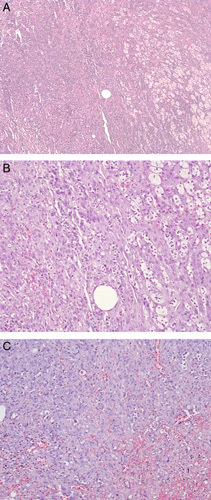
Histological examination. (A) Histological images (4× magnification) of the formalin-fixed paraffin-embedded sections from adrenal gland tissue infiltrated by malignant vascular tumor, that is angiosarcoma. (B) Histological images (10× magnification) of the formalin-fixed paraffin-embedded sections from adrenal gland tissue infiltrated by malignant vascular tumor, that is angiosarcoma. (C) Histological images (10× magnification) of the formalin-fixed paraffin-embedded sections from the tumor showing malignant proliferation formed of intercommunicating vascular spaces lined by malignant epithelioid cells with interstitial hemorrhage.

**Figure 4:**
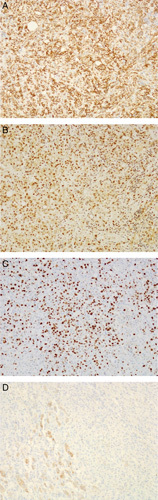
Immunohistochemistry slides. (A) Histological images (10× magnification) of immunohistochemically stained slide of CD31 showing the diffuse positive reaction of tumor cells. (B) Histological images (10× magnification) of immunohistochemically stained slide of FLI-1 showing the diffuse positive reaction of tumor cells. (C) Histological images (10× magnification) of immunohistochemically stained slide of Ki67 showing a high (more than 50%) proliferative index of tumor cells. (D) Histological images (10× magnification) of immunohistochemically stained slide of inhibin showing positive residual adrenal tissue with negative staining of tumor cells.

The excised specimen showed a well-circumscribed encapsulated mass with a size of 14 cm upon gross pathological evaluation. The tumor had invaded through the adrenal capsule and exhibited areas of necrosis and hemorrhage. No residual adrenal tissue was observed, and lymphovascular invasion was identified (Fig. 3). Immunohistochemistry results were positive for pan-cytokeratin, CD31, FLI-1, and D2-40 and negative for chromogranin, synaptophysin, melan-A, inhibin, S100, desmin, and myogenin. These findings were consistent with epithelioid angiosarcoma (Fig. 4).

**Figure 5:**
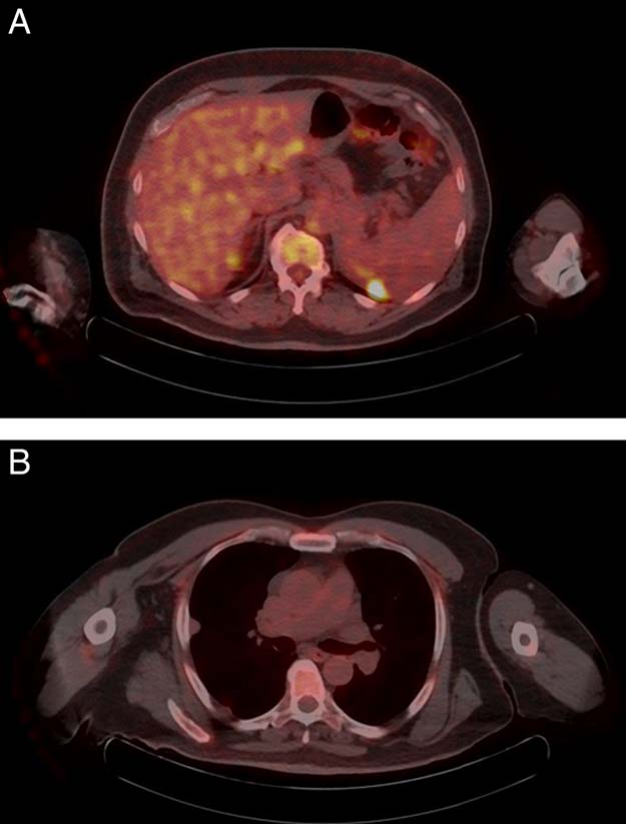
Positron emission tomography images at the time of diagnosis. (A) The mass exhibited minimal nonspecific heterogeneous fluorodeoxyglucose (FDG) activity and measured ~7×4.5 cm in its maximal axial dimensions. (B) The scan revealed multiple bilateral pulmonary nodules throughout both lung fields, showing variable degrees of FDG avidity, suggesting a metastatic process.

**Figure 6:**
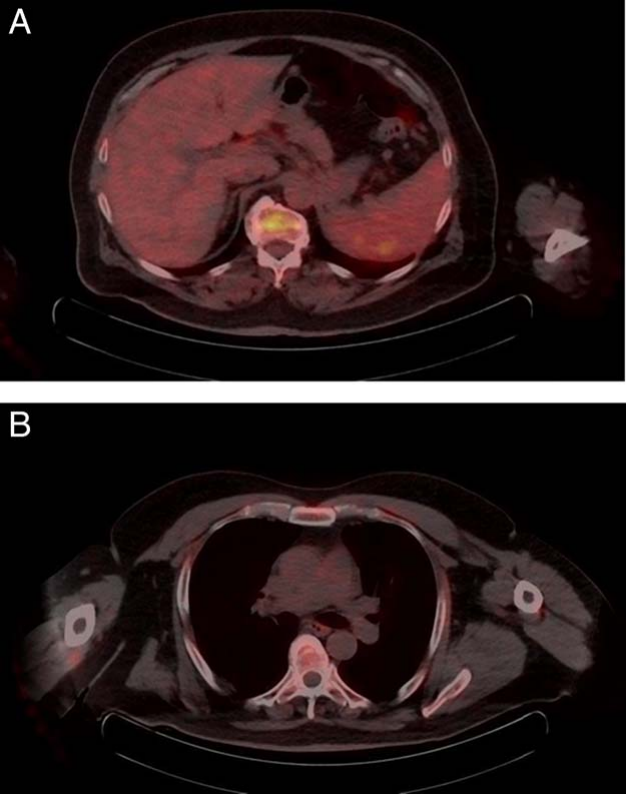
Positron emission tomography after treatment. (A) No definitive hypermetabolic lesion at the anatomical area of the primary lesion. There is slight fluorodeoxyglucose (FDG) activity which is consistent with postoperative changes. (B) Complete resolution of the previously seen hypermetabolic pulmonary nodules.

The patient had an uneventful postoperative course and underwent six cycles of adjuvant chemotherapy with doxorubicin and ifosfamide with good tolerance. A positron emission tomography (PET) scan evaluation performed after completion of the chemotherapy revealed an ill-defined mass at the site of the previous mass with areas of calcification that could not be separated from both the pancreas and the left kidney. The mass exhibited minimal nonspecific heterogeneous fluorodeoxyglucose (FDG) activity and measured ~7×4.5 cm in its maximal axial dimensions (Fig. 5). The scan also revealed multiple bilateral pulmonary nodules throughout both lung fields, showing variable degrees of FDG avidity, suggesting a metastatic process. As a result, the patient was considered to have metastatic disease and started on a new line of chemotherapy consisting of gemcitabine (825 mg/m^2^) and docetaxel (75 mg/m^2^). The patient completed a total of six cycles and underwent a follow-up PET scan that showed no definite hypermetabolic lesion at the surgical bed and complete resolution of the previously seen hypermetabolic pulmonary nodules (Fig. 6). At 11 months postoperatively, the patient was alive and in remission.

## Discussion

Angiosarcoma is a rare malignancy derived from the endothelium of lymphatic or blood vessels^7^, accounting for less than 1% of all sarcomas^3^. The head and neck are the most common sites for this tumor, followed by the breast and extremities^8^. When polygonal endothelial cells with an epithelioid appearance are arranged in solid sheets and nests, epithelioid angiosarcoma develops, a morphological variant of angiosarcoma^1^. Primary adrenal epithelioid angiosarcoma is an infrequent entity. It was first described by Kareti *et al.* in 1998^5^. The etiology of epithelioid angiosarcoma remains unknown, but it has been associated with exposure to many chemicals, including arsenic, vinyl chloride, anabolic steroid therapy, and thorium dioxide^2^. Other predisposing risk factors include familial angiodysplasia, chronic lymphoedema, and radiation^9^. Men are generally more affected than women, with a median age of 60 years^3^.

The most common presentation is an abdominal mass associated with pain, which may be associated with episodes of fever, weight loss, and weakness, although it may be asymptomatic with an incidental finding on imaging^2^. The rarity of this disease makes the diagnosis of this tumor very challenging. In addition, many other differential diagnoses, such as pheochromocytoma, adrenal cortical carcinoma, metastatic carcinoma, lymphoma, and metastatic melanoma, should be considered and ruled out^8^. Furthermore, adrenal adenomas and pheochromocytomas have cystic changes similar to those observed in this tumor^5^.

The size of adrenal angiosarcoma ranges from 5 to 16 cm in diameter, and it grossly varies from solid to cystic, well-circumscribed to invasive retroperitoneal masses^9^.

The tumor appears as a heterogenous mass on CT images, with a hyperattenuating area indicating active hemorrhage or calcification and a hypoattenuating area representing necrosis which does not enhance after contrast^9^.

Histologically, it shows a sheet of large pleomorphic round epithelioid cells with eosinophilic cytoplasm, with prominent nucleoli forming the lining of interlacing vascular spaces^8^. In terms of immunohistochemistry, the epithelioid variant of angiosarcoma stains positive for cytokeratin, which can also be positive in other mesenchymal tumors and metastatic epithelial tumors^5^. Therefore, to avoid misdiagnosis, a broad immunohistochemical panel with positive staining for vascular markers such as ERG, CD31, CD345, and factor 8 is required for definitive diagnosis^2^. In addition, a negative result for melan-A is used as an additional tool to rule out adrenal cortical tumors^10^.

There are no specific imaging findings for this tumor, so histology combined with immunohistochemistry is the most definitive diagnostic method^10^.

Due to the rarity of this disease, treatment options other than surgical excision remain controversial^3^. Surgical excision, which may be adrenalectomy alone or adrenalectomy with splenectomy or nephrectomy, depending on the stage of disease and extent of spread^8^, is the mainstay of treatment.

The management of adrenal angiosarcoma is challenging, with a reported 5-year survival rate of less than 30%^7^. While the use of adjuvant chemotherapy in treating this type of malignancy remains uncertain, some studies suggest that it may improve the 2-year survival rate for patients with visceral angiosarcoma. Currently, there are no evidence-based guidelines for administering adjuvant chemotherapy after complete resection of the primary tumor^8^. However, adjuvant therapy is recommended for patients with incomplete extirpation after surgery, which can be detected by a 3–6-month follow-up interval^11^. Treatment options for local recurrence or distant metastases include chemotherapy or radiotherapy^4^.

Table 1 provides a summary of the age, sex, management, and clinical outcomes of reported cases of adrenal angiosarcoma in the past 5 years. For instance, Case 1 involved a patient with epithelioid angiosarcoma of the adrenal gland in the background of an adrenocortical adenoma. Immunohistochemistry tests were positive for CD31 and erythroblast transformation-specific related gene, with focal staining for pan-cytokeratin in malignant cells. Notably, the patient also had metastatic angiosarcoma in the lungs, as revealed by a PET scan. Following adrenalectomy, the patient received Paclitaxel chemotherapy, resulting in a 14-month survival postoperatively^11^.

**Table 1:** Summary of previously reported cases in the last 5 years with their clinical outcomes

Case #	Age (year)/sex	Size (cm)	Cite	Treatment	Chemotherapy/radiotherapy	Concomitant adrenal lesion	Relation to the adjacent organs/distant metastasis	Clinical outcome
1^11^	44/F	11.7	Left	AE	Chemotherapy (paclitaxel) for metastatic disease	Bilateral adrenal nodules	Metastasis in the lungs	Alive on follow-up 14 months after resection
2^11^	65/M	11.2	Left	AE	None	None	Suspected recurrent angiosarcoma with bowel invasion after 4 months of AE	Died 10 months post-surgery
3^5^	58/M	17.5	Left	AE, left nephrectomy, splenectomy, partial gastrectomy, and distal pancreatectomy	Adjuvant chemotherapy with paclitaxel. Chemotherapy for metastatic disease with doxorubicin, then gemcitabine with vinorelbine, then carboplatin, and finally, etoposide doxorubicin-mitotane	As the disease progression, metastasis to the contralateral adrenal gland	Extensive retroperitoneal metastatic disease, metastasis to the right adrenal gland and distal colon	Died 9 months post-surgery
4^12^	60/M	6.5	Left	AE	None	None	None	Alive, NED at 3 years
5^12^	60/M	8	Left	AE, left nephrectomy and splenectomy	None	Adrenal cortex hyperplasia and cystic lesion	Adhesions to the capsule of the kidney and spleen	Died of disease at 1 month
6^7^	57/M	7.4	Left	AE and splenectomy	None	None	Simultaneous occurrence of angiosarcoma in the spleen	Alive, NED at 18 months
7^13^	38/F	8.5	Right	AE	Chemotherapy for metastatic disease (paclitaxel)	None	Metastasis to ribs and right tibia	Alive, 6 months of post-surgery
8^10^	51/M	4	Left	AE	Chemotherapy for recurrence and metastasis, combination chemotherapy, consisting of six cycles of doxorubicin plus paclitaxel	None	Recurrence after 7 months after surgery, distant metastasis to mediastinal, inguinal, and retroperitoneal lymph nodes	Alive, 12 months post-surgery
Our case	59/M	14	Left	AE	Six cycles of adjuvant chemotherapy (doxorubicin and ifosfamide) For metastatic lesions: six cycles of (gemcitabine and docetaxel)	None	The primary tumor was adherent to adjacent organs but did not invade them. Recurrence in the local area and metastasis to both lungs	Alive, 11 months post-surgery

AE, adenectomy; NED, no evidence of recurrent or metastasis.

Similarly, in Case 6, the patient had synchronous angiosarcoma in the adrenal gland and spleen. Immunohistochemistry tests confirmed the diagnosis, with positive results for CD31, CD34, and factor 8 in both organs. The patient underwent simultaneous adrenalectomy and splenectomy and was alive and in remission 18 months postoperatively^7^.

## Conclusion

In summary, this case report documents an unusual presentation of epithelial angiosarcoma in a 59-year-old male patient. Surgical excision was followed by adjuvant chemotherapy after a detailed evaluation of the immunohistochemistry results. The rarity of this type of malignancy underscores the importance of a team-based approach to ensure the most effective treatment plan is implemented. In cases of rare malignancies, interdisciplinary collaboration is crucial for determining the optimal management strategy. This case highlights the significance of accurate diagnosis, thorough pathological analysis, and prompt treatment of angiosarcomas to achieve positive outcomes.

## Ethical approval

This study is exempt from ethical approval in our intuition.

## Consent

Written informed consent was obtained from the patient for the publication of this case report and accompanying images. A copy of the written consent is available for review by the Editor-in-Chief of this journal on request.

## Source of funding

The study did not receive any financial help.

## Author contribution

M.N.: data collection; M.N., A.M.M.Z., and Z.M.M.Z.: study concept or design; M.N., A.M.M.Z., and Z.M.M.Z.: writing the manuscript; M.N., A.M.M.Z., and Z.M.M.Z.: review and editing the manuscript; M.Q. and M.F.: histopathological interpretation.

## Conflicts of interest disclosure

The authors have no conflicts of interest to declare.

## Research registration unique identifying number (UIN)

The study does not have a trial registry number.

## Guarantor

Dr Yousef Hamamreh.

## Provenance and peer review

Not commissioned, externally peer-reviewed.

## Data availability statement

Not available.
